# The Neural Substrates of Infant Sleep in Rats

**DOI:** 10.1371/journal.pbio.0030143

**Published:** 2005-04-19

**Authors:** Karl Æ Karlsson, Andrew J Gall, Ethan J Mohns, Adele M. H Seelke, Mark S Blumberg

**Affiliations:** **1**Program in Behavioral and Cognitive Neuroscience, Department of PsychologyUniversity of Iowa, Iowa City, IowaUnited States of America; University of California at Los AngelesUnited States of America

## Abstract

Sleep is a poorly understood behavior that predominates during infancy but is studied almost exclusively in adults. One perceived impediment to investigations of sleep early in ontogeny is the absence of state-dependent neocortical activity. Nonetheless, in infant rats, sleep is reliably characterized by the presence of tonic (i.e., muscle atonia) and phasic (i.e., myoclonic twitching) components; the neural circuitry underlying these components, however, is unknown. Recently, we described a medullary inhibitory area (MIA) in week-old rats that is necessary but not sufficient for the normal expression of atonia. Here we report that the infant MIA receives projections from areas containing neurons that exhibit state-dependent activity. Specifically, neurons within these areas, including the subcoeruleus (SubLC), pontis oralis (PO), and dorsolateral pontine tegmentum (DLPT), exhibit discharge profiles that suggest causal roles in the modulation of muscle tone and the production of myoclonic twitches. Indeed, lesions in the SubLC and PO decreased the expression of muscle atonia without affecting twitching (resulting in “REM sleep without atonia”), whereas lesions of the DLPT increased the expression of atonia while decreasing the amount of twitching. Thus, the neural substrates of infant sleep are strikingly similar to those of adults, a surprising finding in light of theories that discount the contribution of supraspinal neural elements to sleep before the onset of state-dependent neocortical activity.

## Abstract 2

Unexpectedly, the anatomy and neurophysiology of brainstem areas associated with sleep in the neonatal rat are strikingly similar to the adult

## Introduction

When compared against an adult standard, infant sleep is composed of a reduced set of components. Perhaps the most notable difference between the sleep of infants and adults is the absence of clearly differentiated and sleep-related neocortical electroencephalographic (EEG) activity [1,2,3]. Nonetheless, sleep and wakefulness in the infants of altricial species, including rats, are reliably characterized, respectively, by periods of myoclonic twitching expressed against a background of muscle atonia and high-amplitude behaviors (e.g., locomotion or stretching) expressed against a background of high muscle tone [4]. Although myoclonic twitching during active sleep (AS) in infants is more prevalent and more intense than that seen during rapid eye movement (REM) sleep in adults, its similarities to the adult behavior and its linkage to periods of atonia suggest developmental continuity between the infant and adult sleep states [4,5,6]. Opinions differ, however, as to whether these similarities provide sufficient evidence that infant sleep is developmentally continuous with that of adults. Specifically, it has recently been argued that sleep during the “pre-EEG” period—that is, before postnatal day 12 (P12) in rats [2]—is best considered a primitive state, comprising an amalgam of sleep components, that only gradually differentiates into the distinct sleep states characteristic of adults [1,7].

Proponents of the “amalgam” theory of sleep development have made strong claims concerning the neural substrates of infant sleep. For example, it has been argued that infant sleep is (i) undifferentiated and (ii) controlled by distinct neurophysiological mechanisms from those that generate sleep in adults. In particular, Adrien [8] writes, “In the immediate postnatal period, and furthermore in utero, AS would seem to be very different—even in terms of underlying mechanisms—from what we know of the sleep phenomenon in the adult” (p. 40). Extending this idea, Adrien and Lanfumey [9] state that neural activity during infant sleep indicates “a very primitive system of diffuse activation within the whole central nervous system” (p. 7). More recently, Frank and Heller [1] write, “Brainstem-midbrain nuclei important in mediating REM sleep expression do not mediate the expression of AS, or AS myoclonia” (p. 64). Although several papers have reported state-dependent neural activity within the brainstem of infant rats [10,11], they have not proved convincing [7].

Despite the absence of well-differentiated EEG activity in rats before P12, it is now apparent that the state characterized by nuchal atonia—with or without the simultaneous occurrence of myoclonic twitching—satisfies many established criteria of sleep [12,13,14]. For example, during infant sleep, sensory thresholds increase, both under normal conditions [15] and during sleep deprivation [16]; infant sleep is also homeostatically regulated [16]. In addition, it was recently shown that there exists an inhibitory area within the ventromedial medulla, the medullary inhibitory area (MIA), that appears functionally identical to the corresponding area that mediates REM-sleep atonia in adults [17]. The MIA (i) causes atonia when stimulated; (ii) contains neurons that exhibit atonia-related discharge profiles; and (iii) when lesioned, results in the partial loss of atonia and the decoupling of the components of infant sleep (i.e., “REM sleep without atonia”). The MIA is not sufficient, however, for generating infant sleep, because decerebrations rostral to the MIA, but caudal to the mesopontine region, abolish sleep-wake cyclicity and reduce myoclonic twitching [18] (Karlsson and Blumberg, unpublished data). Thus, expression of the components of infant sleep—atonia and twitching—depends on a network that spans the mesopontine-medullary region.

The aims of the current study were to identify the structures that project to the MIA (and therefore may contribute to the generation of atonia) and to identify supraspinal neural correlates of myoclonic twitching. First, we performed retrograde tracing from the MIA as well as the ventrolateral medulla (an area that typically induces motor activity when stimulated [17]). Next, using the tracing data as a guide, we conducted neural recording studies to determine the relationship, if any, between the discharge profiles of individual neurons and the specific components of infant sleep. Finally, we lesioned structures containing neurons that exhibit opposing state-dependent discharge profiles and examined the effects on behavioral states. These experiments provide the most comprehensive survey to date of the neural control of sleep in infancy and reveal surprising parallels between the neural substrates of sleep in infant and adult rats. Moreover, these results are not consistent with the perspective that views infant sleep as “diffuse,” “undifferentiated,” or arising from “distinct neurophysiologic mechanisms” [1,7,8,9]. Finally, the current findings provide a guide to future theorizing about the ontogeny and function of sleep.

## Results

### Mesopontine and Medullary Structures Project to the Medullary Inhibitory Area

In order to reveal the afferent structures to the MIA that may be important for the expression of muscle atonia, the retrograde tracer DiI was infused into the medial medulla in 5 P8 rats (Figure 1). Medial DiI infusions resulted in light but widespread labeling throughout the pontomedullary axis. Within the medulla, the giant cells of nucleus gigantocellularis (Gi) were consistently labeled (Figure 2A), as were neurons in nucleus magnocellularis and nucleus paramedianis. The vestibular nuclei and cholinergic C3 were lightly labeled and the nucleus of the solitary tract was moderately labeled. Light to moderate labeling was also found along the longitudinal axis from Gi through the nucleus pontis caudalis (PC) to the nucleus pontis oralis (PO). Within the pons, clusters of labeled neurons were consistently found ventral to the locus coeruleus (LC) in the subcoeruleus (alpha, dorsal, and ventral parts, hereafter designated SubLC; Figure 2B). Neurons associated with the midline raphé system (i.e., raphé obscurus and medial and dorsal raphé, DR) were heavily labeled (Figure 2C). Finally, neurons of the dorsal pontine tegmentum, including the laterodorsal tegmental nucleus (LDT; Figure 2D), the pendunculopontine tegmental nucleus (PPT), and the periaqueductal gray were lightly, but consistently, labeled.

**Figure 1:**
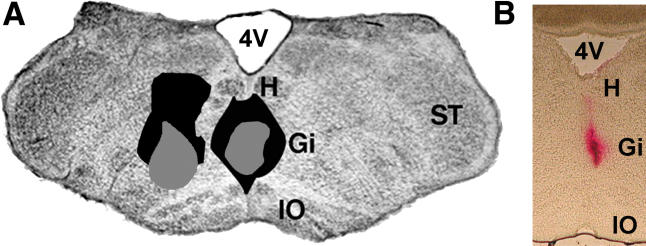
DiI Infusion Sites (A) Halos from 20 nl medial (*n* = 5) and lateral (*n* = 5) DiI infusions recreated from fluorescent photomicrographs on a coronal section of the medullary inhibitory area of a P8 rat. The largest (black) and smallest (gray) halos are shown. (B) Light photomicrograph of a representative DiI infusion site in the medial medulla. 4V, fourth ventricle; H, hypoglossal nucleus; Gi, nucleus gigantocellularis; IO, inferior olive; ST, spinal trigeminal nucleus

**Figure 2:**
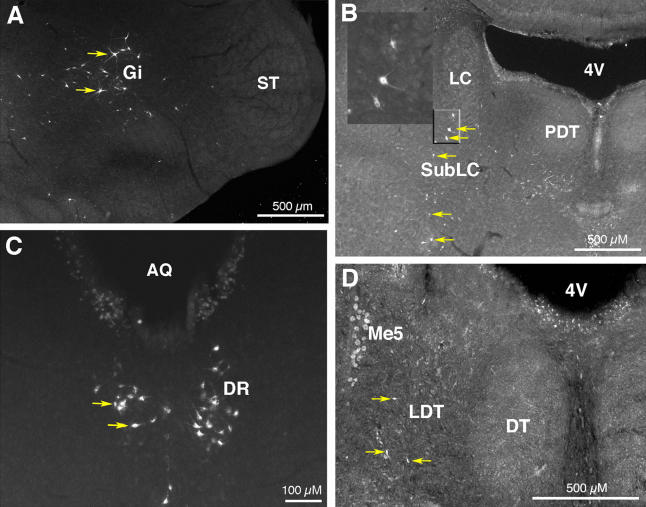
Representative Labeling of Cell Bodies in Selected Areas after a DiI Infusion into the Medullary Inhibitory Area The arrows indicate examples of labeled neurons in (A) nucleus gigantocellularis (Gi), (B) subcoeruleus (SubLC), (C) dorsal raphé (DR), and (D) laterodorsal tegmental nucleus (LDT). Inset in (B) depicts enlarged view of the boxed area. 4V, fourth ventricle; AQ, cerebral aqueduct; DT, dorsal tegmental nucleus; Me5, mesencephalic trigeminal nucleus; ST, spinal trigeminal nucleus

Because our previous study [17] had indicated that motor facilitatory neurons were typically found lateral to the MIA, DiI was also infused laterally to the MIA in five additional P8 rats. Overall, few differences in labeling were observed between the medially and laterally infused groups, with the exception that neurons associated with the raphé system were labeled only after medial infusions.

After visual inspection of the tracing data, seven medullary and pontine structures known to modulate behavioral state regulation in adults (the Gi, PC, SubLC, LC, PO, LDT, and DR) were selected for analysis. The cell counts for these seven structures are summarized in Table 1.

**Table 1:**
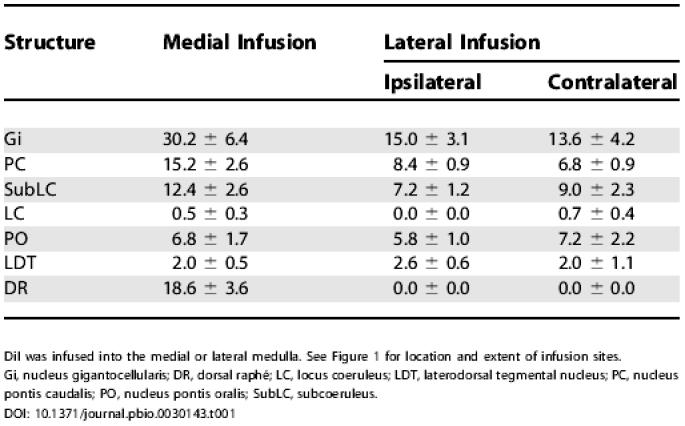
Mean Number (± Standard Error) of DiI-Labeled Neurons within Seven Medullary and Mesopontine Structures in P8 Rats

DiI was infused into the medial or lateral medulla. See Figure 1 for location and extent of infusion sites

Gi, nucleus gigantocellularis; DR, dorsal raphé; LC, locus coeruleus; LDT, laterodorsal tegmental nucleus; PC, nucleus pontis caudalis; PO, nucleus pontis oralis; SubLC, subcoeruleus

### Extracellular Recordings Reveal State-Dependent Neuronal Activity in Medullary and Mesopontine Structures

A total of 142 neurons were recorded from 25 P6–P10 rats (1–6 neurons per rat; 1–4 neurons per recording site) with precollicular decerebrations. (Although precollicular decerebrations disconnect the brainstem from rostral structures known to regulate sleep and wakefulness, infants nonetheless exhibit coherent organization of sleep-wake states; the most noticeable change induced by these decerebrations is more rapid cycling between sleep and wakefulness [19].) Fifty-two neurons did not exhibit any state dependency and were not analyzed further. Five neurons were excluded due to unclear histology. The remaining 85 neurons were assigned to one of five classes on the basis of the specific relationship of the unit activity to the components of sleep and wakefulness (Figure 3A). The interrelations among the classes, and the number of neurons found within each class, are depicted in Figure 3B.

**Figure 3:**
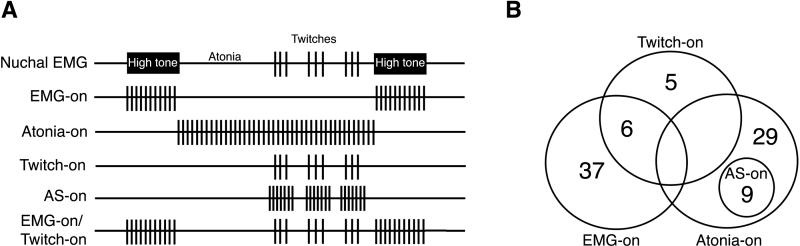
Schematic Representation of the Classes of Neurons Identified in the Current Study and Their Prevalence (A) Summary of the classes of discharge profiles described in the present study in relation to nuchal EMG activity. (B) Venn diagram depicting the interrelation of the classes of state-dependent neurons. Values indicate the number of neurons in each class found in the present study.

### The Lateral Medulla Contains Both EMG-On and Atonia-On Neurons

A total of 13 state-dependent neurons were recorded in the medulla, all within the Gi (Figure 4A). Eight neurons exhibited significantly lower discharge rates during periods of atonia (indicative of sleep) than during periods of high muscle tone (indicative of wakefulness; discharge rates, 1.74 ± 0.38 Hz during atonia; 3.79 ± 0.60 Hz during high tone), and five neurons exhibited significantly higher discharge rates during atonia than high tone (discharge rates, 1.21 ± 0.40 Hz during atonia; 0.76 ± 0.29 Hz during high tone). Representative single unit activity and concurrently recorded electromyograms (EMGs) are depicted in Figures 4C and 4D. Average spike durations of atonia-on and EMG-on neurons were 1.87 ± 0.01 ms and 1.74 ± 0.07 ms, respectively (a single-factor ANOVA revealed no significant group differences in spike durations between any of the classes of neurons reported in the current study).

**Figure 4:**
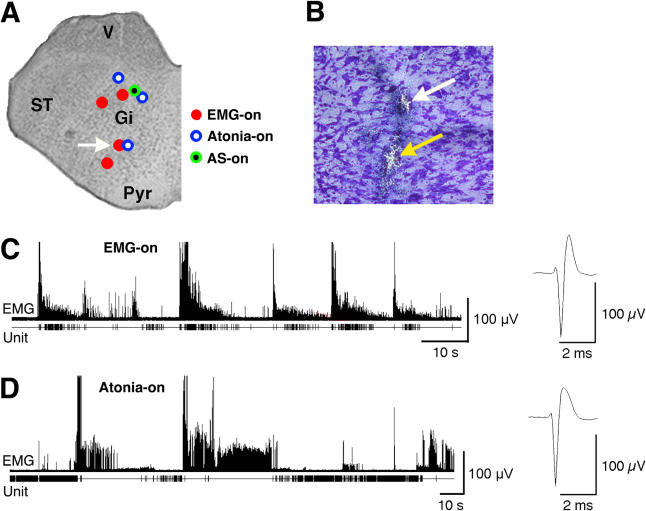
State-Dependent Neural Activity within Nucleus Gigantocellularis (A) Recording sites of state-dependent neurons reconstructed on a coronal section of the medulla of a P8 rat. Note the anatomical overlap of the different classes of neurons. Arrow corresponds to top arrow in (B). (B) Photomicrograph depicting two marking lesions in nucleus gigantocellularis (the two lesions are approximately 80 μm apart). The white arrow indicates the location of the recording site identified by the arrow in (A). (C) Lower trace: single unit activity of a representative Gi EMG-on neuron. Upper trace: concurrently recorded nuchal EMG. Far right: Averaged waveform of representative EMG-on neuron. (D) Lower trace: single unit activity of a representative Gi atonia-on neuron. Upper trace: concurrently recorded nuchal EMG. Far right: Averaged waveform of representative atonia-on neuron. Gi, nucleus gigantocellularis; Pyr, pyramids; ST, spinal trigeminal nucleus; V, vestibular nucleus

### The Subcoeruleus Contains a High Concentration of Atonia-On Neurons

Of a total of 38 neurons classified as atonia-on, 17 were located within the SubLC (discharge rates, 2.64 ± 0.48 Hz during atonia; 0.75 ± 0.23 Hz during high tone); EMG-on neurons were not found within the SubLC. Within the brainstem, atonia-on neurons were also recorded in the dorsomedial tegmental nucleus, ventral tegmental nucleus, and the medial and lateral parabrachial nuclei (MPB and LPB, respectively; see Figure 5A). Spike durations ranged from 1.46 ± 0.75 ms for atonia-on neurons in the LPB to 1.87 ± 0.03 ms for atonia-on neurons in the SubLC. The averaged spike waveform of a representative atonia-on neuron recorded within the SubLC is depicted in Figure 5B. Typically, neurons that were classified as atonia-on discharged tonically throughout the atonia period (Figure 5C).

**Figure 5:**
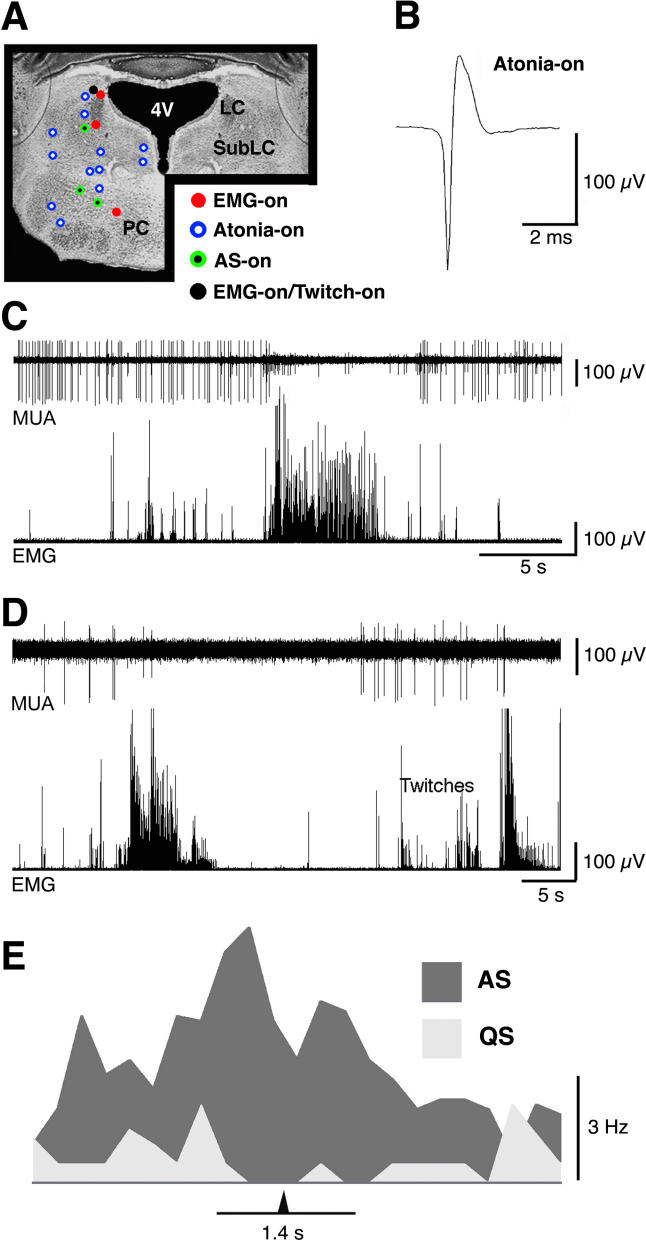
State-Dependent Neural Activity within the Mesopontine Region (A) Recording sites of state-dependent neurons reconstructed on a coronal section at the mesopontine level of a P8 rat. Note the predominance of atonia-on neurons. (B) Averaged waveform of a representative atonia-on neuron. (C) Upper trace: multiunit activity. Lower trace: concurrently recorded nuchal EMG. Spike sorting revealed two units that are easily distinguished by their amplitudes. The higher-amplitude unit is atonia-on; note its tonic discharge throughout the atonia period. (D) Upper trace: multiunit activity. Lower trace: concurrently recorded nuchal EMG. Spike sorting revealed two units that are easily distinguished by their amplitudes. The higher-amplitude unit is AS-on; note the absence of multiunit activity at the onset of the atonia period and then the increase in activity coinciding with the appearance of nuchal twitches. (E) Mean discharge rates of a representative AS-on neuron during bouts of AS and QS as defined, respectively, by the presence or absence of phasic nuchal twitches during periods of atonia. The arrowhead indicates the midpoint of the AS and QS bouts. 4V, fourth ventricle; LC, locus coeruleus; PC, nucleus pontis caudalis; SubLC, subcoeruleus;

### A Subset of Atonia-On Neurons Discharge at Higher Rates during Active Sleep

In contrast to the neuron depicted in Figure 5C, nine of the atonia-on neurons exhibited significantly higher discharge rates during AS, defined as periods of atonia accompanied by bouts of twitches of the tail and nuchal muscle. These neurons were classified as AS-on (see Materials and Methods). A representative recording of multiunit activity that includes an AS-on neuron is depicted in Figure 5D. In Figure 5E the average discharge rate of a representative AS-on neuron during AS and QS (defined as periods of atonia without myoclonic twitching) is depicted.

Four neurons recorded within the SubLC were AS-on (discharge rates, 1.84 ± 0.67 Hz during AS; 0.73 ± 0.28 Hz during QS); three medullary neurons were AS-on (discharge rates, 1.99 ± 0.60 Hz during AS; 0.91 ± 0.30 during QS); and two neurons within the MPB were AS-on (discharge rates, 0.43 ± 0.02 Hz during AS; 0.09 ± 0.02 Hz during QS). Spike widths of AS-on neurons ranged from 1.95 ± 0.10 ms in the SubLC to 2.03 ± 0.16 ms in Gi.

### The Infant Locus Coeruleus Contains EMG-On Neurons

Neurons exhibiting EMG-on profiles were recorded in the LPB, PC, and the LC. Within the LC (Figure 6A), two such units were found. The averaged spike waveform for one of these units is depicted in Figure 6B (both neurons exhibited short spike durations, 1.76 ± 0.12 ms). As shown in Figure 6C, both LC units increased their discharge rates primarily during periods of high muscle tone and were virtually silent during periods of atonia (discharge rates, 0.38 ± 0.17 during atonia; 7.81 ± 0.06 Hz during high tone), apart from rare bursts of activity during atonia (e.g., as indicated by the arrow in Figure 6C).

**Figure 6:**
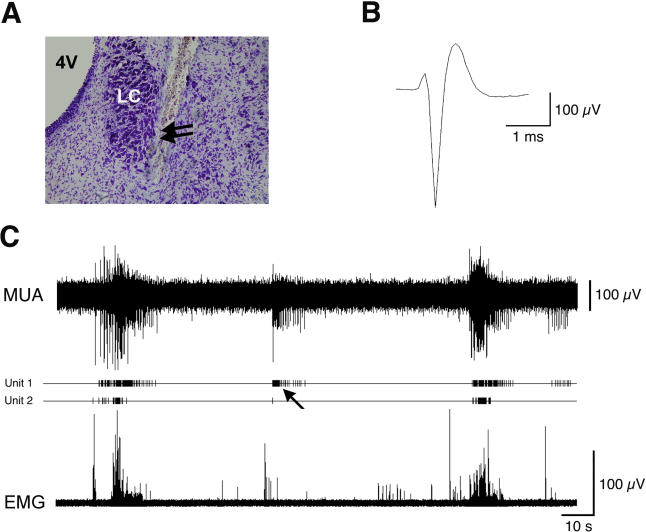
Activity of LC Neurons across the Sleep-Wake Cycle in a P8 Rat (A) Coronal section of the LC indicating the recording site (double arrows). (B) Averaged waveform of unit 2 in (C). (C) Upper trace: LC multiunit activity. Middle traces: Activity of two isolated units derived from the multiunit activity. Bottom trace: concurrently recorded nuchal EMG. 4V, fourth ventricle; LC, locus coeruleus

### The Dorsolateral Pontine Tegmentum Contains EMG-On Neurons

In Figure 7A, the recording sites within the pontine tegmentum are depicted. Seventeen of 37 EMG-on neurons were located within a small region of the dorsolateral pontine tegmentum that includes LDT, the cuneiform nucleus, and periaqueductal gray; the remainder were distributed within the LC (see Figure 6), PC (see Figure 5), and Gi (see Figure 4). An averaged spike waveform of a representative EMG-on neuron, recorded in LDT, is depicted in Figure 7B (mean spike durations of EMG-on neurons ranged from 1.62 ± 0.11 ms in the LDT to 2.36 ± 0.14 ms in the PC) and a representative recording of multiunit activity that includes an EMG-on neuron is depicted in Figure 7C. The discharge rates of non-LDT EMG-on neurons ranged from 0.66 ± 0.40 Hz during atonia in the PC to 4.75 ± 1.09 Hz during high tone in the cuneiform nucleus. The relatively high discharge rates (5.67 ± 1.49 Hz during atonia and 11.10 ± 2.22 Hz during high tone) and short spike durations of LDT EMG-on neurons suggest that they may be non-cholinergic [20].

**Figure 7:**
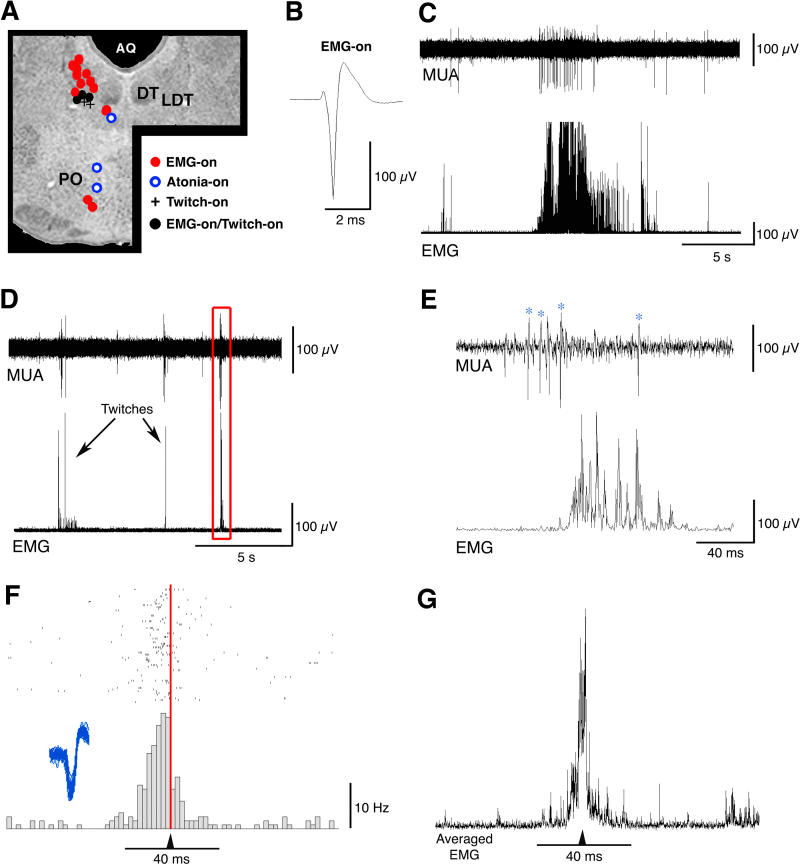
State-Dependent Neuronal Discharges within the Pontine Tegmentum (A) Recording sites of state-dependent neurons reconstructed on a coronal section of the brainstem. Note the predominance of EMG-on neurons. (B) Averaged waveform of a representative EMG-on neuron. (C) Upper trace: multiunit activity. Lower trace: concurrently recorded nuchal EMG. One EMG-on neuron was isolated from the multiunit record; note its tonic discharge during the period of high muscle tone. (D) Upper trace: multiunit activity. Lower trace: concurrently recorded nuchal EMG. (E) Expanded view of the boxed area from (D). Note how multiunit activity precedes the twitch. Asterisks identify a single isolated unit. (F) Peristimulus histogram and raster plot for the twitch-on neuron identified in (E) during a 10-min recording session in a P7 rat (83 total twitches). Inset depicts 55 superimposed action potential waveforms for this unit. This unit's mean discharge rate peaks 5–10 ms before the twitch (red line). (G) Averaged nuchal EMG for all 83 twitches represented in (F). AQ, cerebral aqueduct; DT, dorsal tegmental nucleus; LDT, laterodorsal tegmental nucleus; PO, nucleus pontis oralis

### The Laterodorsal Tegmental Nucleus Contains Twitch-On Neurons

The LDT contains neurons that exhibit a burst of action potentials in anticipation of myoclonic twitches (designated as twitch-on neurons). Ten of the 11 recorded neurons that exhibited this activity pattern were found within the LDT (Figure 7A); one neuron was located in the parabrachial nucleus (see Figure 5A). Six of these neurons were both EMG- and twitch-on (designated as EMG-on/twitch-on); that is, they exhibited higher discharge rates during periods of high muscle tone than during periods of atonia (mean discharge rates, 15.92 ± 5.93 during high tone; 6.80 ± 5.61 during atonia) while also firing in anticipation of myoclonic twitches (see below). In addition, the EMG-on/twitch-on neurons had slightly broader average spike durations than did twitch-on neurons (1.92 ± 0.13 ms and 1.63 ± 0.16 ms, respectively).

Over the 60-ms period before a myoclonic twitch, the mean discharge rate of twitch-on neurons increased dramatically, from 2.81 ± 0.87 Hz to 24.22 ± 6.67 Hz. In Figure 7D, a representative recording of multiunit activity that includes a twitch-on neuron is depicted, and the tight coupling of the bursts of multiunit activity and nuchal twitches is apparent. Figure 7E depicts an expanded view of the boxed area from Figure 7D and identifies (with asterisks) an isolated twitch-on unit. Figure 7F depicts a peristimulus histogram and raster plot of an identified unit (the same unit depicted in Figure 7E) triggered on nuchal EMG spikes. Immediately preceding the onset of a twitch, the discharge rate increased more than 30-fold, suggesting that this neuron participates in the production of twitching. It is important to note, however, that the relationship between neuronal discharges and twitches was not perfect; that is, there were occasions when the neuron discharged and a nuchal twitch was not detected, and there were occasions when a nuchal twitch was detected but the neuron did not discharge.

### Lesions of Subcoeruleus and Nucleus Pontis Oralis Reduce Atonia Durations and Produce “REM Sleep without Atonia”

As described above, the SubLC contains a high concentration of atonia-on neurons (see Figure 5); in addition, both the present study (see Figure 7) and that of Corner and Bour [10] suggest that the PO is important for regulating infant sleep. To examine the causal roles played by these areas in sleep and wakefulness, both areas were lesioned here using high concentrations (50 mM) of quisqualic acid, a glutamate receptor agonist (Figure 8).

**Figure 8:**
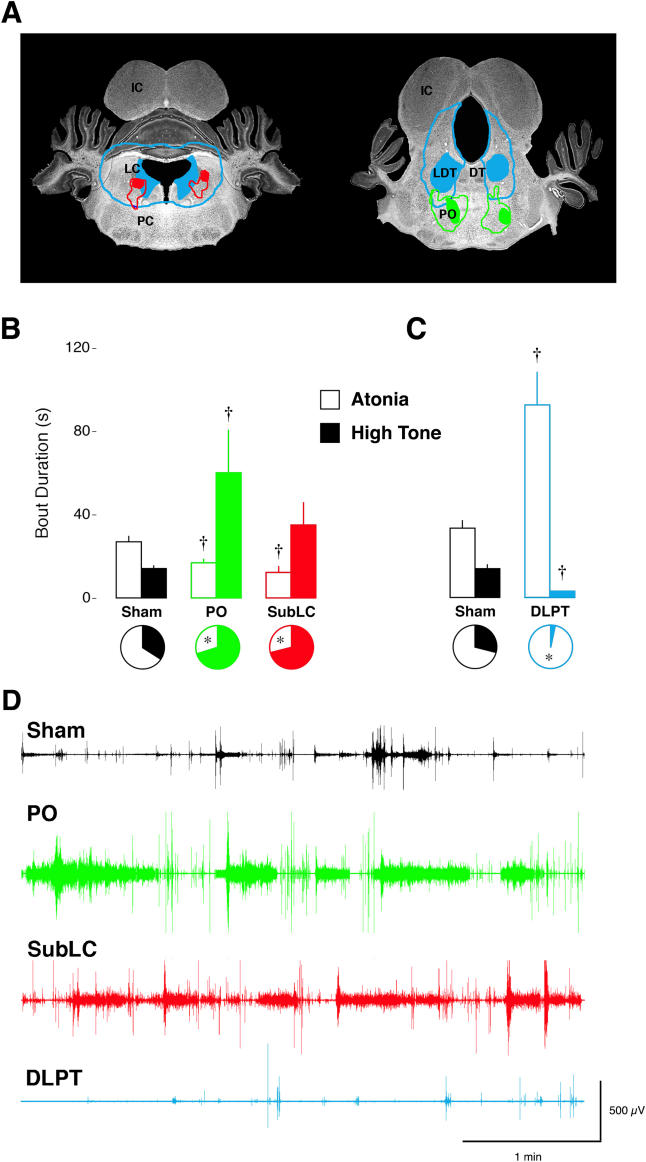
Effects of Brainstem Lesions on the Expression of Nuchal Muscle tone in P8 Rats (A) Representations of lesions of the dorsolateral pontine tegmentum (DLPT; blue), pontis oralis (PO; green), and subcoeruleus (SubLC; red) on coronal sections. Outlined areas indicate the extent of all lesions in a group, and filled areas indicate the smallest lesion in a group. (B) Mean ± standard error bout durations of atonia and high muscle tone for sham (*n* = 7; black), PO lesion (*n* = 5; green), and SubLC lesion (*n* = 4; red) groups. **†** Significant difference from sham group, *p* < 0.05. Pie charts beneath each bar graph indicate percentage of time spent in atonia or high muscle tone. * Significant difference from sham group, *p* < 0.05. (C) Mean ± standard error bout durations of atonia and high muscle tone for sham (*n* = 7; black) and DLPT lesion (*n* = 6; blue) groups. **†** Significant difference from sham group, *p* < 0.05. Pie charts beneath each bar graph indicate percentage of time spent in atonia or high muscle tone. * Significant difference from sham group, *p* < 0.05. (D) Representative 4-min recordings of nuchal EMG for a sham pup and for pups with PO, SubLC, and DLPT lesions. DT, dorsal tegmental nucleus; IC, inferior colliculus; LC, locus coeruleus; LDT, laterodorsal tegmental nucleus; PC, nucleus pontis caudalis; PO, nucleus pontis oralis

The extent of the lesions of the SubLC (*n* = 4) and PO (*n* = 5) is depicted in Figure 8A. As shown in the bar graphs of Figure 8B, these lesions produced significant changes in the mean bout durations of atonia and high muscle tone (atonia, *F*[2,13] = 10.02, *p* < 0.01; high tone, *F*[2,13] = 4.07, *p* < 0.01). Moreover, as indicated by the pie charts in Figure 8B, the percentage of time in atonia was significantly reduced in the lesion groups (*F*[2,13] = 14.2, *p* < 0.0005). Representative recordings of nuchal EMG in pups with SubLC and PO lesions are shown in Figure 8D.

Lesions of SubLC and PO also disrupted the expression of myoclonic twitching of the limbs and tail. Although the absolute number of myoclonic twitches during the 15-min scoring period did not differ between the lesion and sham groups (*F*[2,13] = 0.79, not significant), lesions of SubLC and PO resulted in significant increases in the proportion of twitches that occurred against a background of high muscle tone (*F*[2,13] = 7.91, *p* < 0.01). Specifically, in the SubLC and PO lesion groups, respectively, 27.1% and 16.1% of all twitches were expressed against a background of high muscle tone, in contrast with only 0.01% in the sham group.

### Lesions of the Dorsolateral Pontine Tegmentum Increase Atonia Durations and Suppress Myoclonic Twitching

Because the DLPT contained a high concentration of EMG-on and twitch-on neurons (see Figure 7), and because of difficulties using chemical lesion techniques in this area (see Materials and Methods), electrolytic lesions of the DLPT were performed; the extent of the lesions is shown in Figure 8A. As shown in the bar graphs of Figure 8C and in contrast to lesions of SubLC and PO, lesions of the DLPT produced a sizable increase in the mean bout duration of atonia (*t* = 6.7, *df* = 11, *p* < 0.01) and a decrease in the mean bout duration of high muscle tone (*t* = 3.3, *df* = 11, *p* < 0.01). Consequently, as indicated by the pie charts in Figure 8C, the percentage of time in atonia was significantly increased in the lesion group (*t* = 6.4, *df* = 11, *p* < 0.0001). A representative recording of nuchal EMG in a pup with a DLPT lesion is shown in Figure 8D.

In contrast with lesions of SubLC and PO, DLPT lesions significantly reduced the number of twitches. In the sham group, the mean number of limb twitches over the 15-min period was 518.3 ± 34.3, compared to 302.9 ± 51.7 in the lesion group (*t* = 3.3, *df* = 11, *p* < 0.01). Also, in contrast to the SubLC and PO lesion groups and similar to shams, less than 0.1% of twitches in pups with DLPT lesions were observed during periods of high muscle tone.

## Discussion

In the present study, we outline, to our knowledge for the first time, the neural substrates of two primary sleep components in week-old rats. First, we confirm that the inputs to the MIA from medullary and mesopontine structures are similar to those reported in adults [21,22,23]. Second, neurons exhibiting atonia-on discharge profiles (indicative of sleep) were found predominantly within the SubLC, and neurons exhibiting EMG-on discharge profiles (indicative of wakefulness) were found predominantly within the LDT. Third, we report the presence of neurons that exhibit a distinct bursting pattern that anticipates myoclonic twitches. Fourth, consistent with the recording data, we demonstrate that atonia durations are decreased after lesions of SubLC and PO, and myoclonic twitching is reduced after lesions of the DLPT despite a dramatic increase in atonia duration. Finally, lesions of SubLC and PO decoupled myoclonic twitching from nuchal atonia, producing a condition that resembles REM sleep without atonia as described in juvenile and adult rats [24,25].

The present study adds critical new evidence in support of the notion that sleep in infant rats satisfies most conventional criteria for defining sleep, including state-dependent neural activity [13,14]. This study also supports the contention that the development of sleep in mammals entails the continuous elaboration of components that are identifiable in early infancy [5,6,26,27,28]. These experiments are particularly significant in light of the fact that they were performed using an altricial species at ages before the onset of sleep-related neocortical activity [1,2,3]. Consequently, these results may prove to be broadly applicable to our understanding of sleep mechanisms in other mammalian species at an analogous time in development, including the period before 120 d postconception in sheep [29,30], 50 d postconception in guinea pigs [31], approximately 32 wk postconception in preterm human infants [32], and P13–15 in kittens [28].

We did not find state-dependent activity in the DR despite its heavy labeling after infusions of DiI into the MIA. Nonetheless, it is possible that the DR does contain state-dependent neurons at the ages studied here. For example, it is possible that cessation of DR activity during sleep depends on histaminergic input from the tuberomammillary nucleus [33], a region that was disconnected by the precollicular decerebrations used here (see Materials and Methods). Conversely, within the sparsely labeled LDT, we found neurons that exhibited state-dependent discharge profiles.

The medulla of adults contains neurons that participate in the generation of both sleep and wake phenomena [34]. In adult cats, classes of medullary neurons have been identified that discharge during wakefulness, during REM sleep [35,36,37], and during REM sleep as well as waking movements [35,38,39]. Medullary neurons with similar discharge profiles have also been identified in adults rats [40,41]. In the present study, we found medullary EMG-on neurons that compare well with the wake-on neurons reported in adult rats [40,41] and cats [38,39], atonia-on neurons that compare well with the REM-sleep specific neurons reported by Kanamori et al. [36], and AS-on neurons that compare well with the phasic REM sleep neurons reported by Steriade et al. [35]. It should be noted that we did not find AS-on neurons in the brainstem that exhibited high, tonic rates of discharge typical of some REM-on neurons in adults [33]. More extensive sampling will be necessary to describe fully the activity patterns exhibited by AS-on neurons in infants.

To our knowledge, LC recordings have not been reported previously in unanesthetized infant rats. Moreover, the EMG-on profile of the LC units described here is consistent with their wake-active profile in adult rats [42]. Interestingly, this LC activity profile is consistent with the notion that increased olfactory sensory thresholds during infant sleep result from a decrease in excitatory LC input to mitral cells of the olfactory bulbs [15,43].

In adult cats, REM sleep depends on the integrity of the peri-LC-α, which corresponds to the sublaterodorsal nucleus (SLD) in rats [33,44,45] and is contained within the region designated as the SubLC here. In a recent study aimed at elucidating the neural substrates of sleep in adult rats, Boissard et al. [44] demonstrated that REM sleep can be induced by increasing SLD activity, either by decreasing GABAergic inhibition or by increasing glutamatergic excitation. Moreover, it was demonstrated that the SLD projects to neurons in the medial medulla that also stain positively for glycine [44].

The current study supports and amplifies the findings of Boissard et al. [44]. First, we did not find any neurons within the SubLC that exhibited an EMG-on profile, and the SubLC contained 17 of the 29 atonia-on neurons reported here. Second, using DiI, we demonstrated that the SubLC connects directly with the MIA. Third, chemical lesions of the SubLC significantly reduced atonia durations. Finally, we note that excitation of the infant MIA with a glutamate agonist is sufficient to produce atonia [17]. All together, these findings are consistent with the notion that atonia in infant and adult rats is produced in part by glutamatergic excitatory input from the SubLC to glycinergic neurons of the ventromedial medulla that, in turn, hyperpolarize spinal motoneurons [46,47].

Although lesions of both the SubLC and PO dramatically reduced the duration of atonia periods, they did not reduce rates of myoclonic twitching. It is not surprising, then, that there were many instances when twitches were observed during periods of high muscle tone, a decoupling of tonic and phasic sleep components that resembles REM sleep without atonia produced in adults after pontine [24] or SubLC [48] lesions.

The increase in atonia and the reduction in twitching after DLPT lesions may have resulted, respectively, from the disinhibition of atonia facilitatory neurons in the reticular formation (perhaps within the PO) and the loss of twitch-on neurons within the LDT. Of course, it is also possible that these electrolytic lesions exerted their effects by destroying nearby structures or fibers of passage. We do know, however, that although the DLPT lesions were large and frequently included the LC and SubLC, the effect of the DLPT lesions on atonia and twitching cannot be attributed to damage to either of those two areas, because electrolytic lesions of the LC [19] and chemical lesions of the SubLC (as shown in the present study) reduced mean atonia durations without affecting twitching. Thus, after large DLPT lesions, the PO may contribute to the elongation of atonia periods via direct and mutually facilitatory interactions with the MIA [49].

Previous work in adult cats has explored the importance of the cholinergic mesopontine tegmentum in REM sleep generation [50,51]. The present findings are consistent with this earlier work (although it should be stressed that we have not demonstrated here that these infant mesopontine mechanisms utilize acetylcholine). Webster et al. [50] reported significant decreases in REM sleep after large lesions that destroyed the majority of cholinergic neurons within the LDT, PPT, the parabrachial nucleus, and the LC. Shouse and Siegel [51] demonstrated that lesions primarily involving the PPT resulted in the loss of phasic sleep components, whereas lesions of the SubLC resulted in REM sleep without atonia. In contrast, lesions primarily localized to the LDT and LC did not alter the expression of REM sleep components. Recently, it was reported that discrete PPT lesions in adult rats did not affect sleep time but did interfere with sleep propensity after sleep deprivation [52]; unfortunately, phasic sleep events were not recorded in that study.

In adult rats, REM sleep induced by selective activation of the SLD lacks phasic components (e.g., rapid eye movements) [44]. The authors of that study hypothesized that activation of the phasic components of REM sleep requires activation of neurons within the LDT, presumably in the form of burst-pause discharges such as those identified previously in reticular neurons in adult cats [38,39]. The presence of such discharges in adult rats, however, has been questioned [53]. Here we demonstrate that the LDT (and the parabrachial nucleus) contains twitch-on neurons that appear to participate in the generation of myoclonic twitching of the limbs and nuchal muscle. The burst-pause discharge profile of these twitch-on neurons compares well with neurons recorded in the pontine tegmentum of adult cats (located among fibers of the brachium conjunctivum) that exhibit sharp bursts that anticipate pontine-geniculo-occipital waves [54]. In this regard, it may be significant that LDT neurons burst after activation of the low-threshold calcium current [55,56,57], thus allowing them to behave as single-cell oscillators [58]. We have here, then, a mechanism by which LDT neurons can produce phasic events such as myoclonic twitches. The reduced rates of twitching after DLPT lesions, as shown here, is consistent with this view.

The bursting of a twitch-on neuron was not always associated with a nuchal muscle twitch. One possible explanation for this is that the downstream effects of the activity of twitch-on neurons are gated by the moment-to-moment levels of glycinergic inhibition provided by descending axons from the MIA [17,46,47]. It is also possible that a burst of activity by twitch-on neurons serves to momentarily override the hyperpolarization of spinal motoneurons and thus allows spinal networks to exhibit behaviorally observable spontaneous activity [59].

It has recently been suggested that myoclonic twitches in infants contribute to the self-organization of neural circuits within the spinal cord that govern withdrawal responses to aversive stimuli [60]. Specifically, it was argued that a limb movement during a twitch provides sensory feedback that helps to calibrate interactions among neurons within a reflex module; according to this hypothesis, the temporal correlations among motor and sensory events modify synapses through a Hebbian-type learning process. Indeed, we note that the information generated by the feedback from a single limb twitch against a background of muscle atonia represents a situation that is well suited for Hebbian learning to occur [61]. But this idea may also apply to any myoclonic twitch, whether produced by phasic activation of limb muscles, nuchal muscle, or eye muscles; in fact, phasic activity during sleep in infants is a global phenomenon occurring as temporally coherent bouts of activity in many muscle groups throughout the body [62]. Therefore, we believe that myoclonic twitching will prove to play a broad role in early motor and somatosensory development [26,63].

Whereas the ontogeny of mammalian sleep remains a topic of dispute [7,14], some aspects of that debate can now be put to rest. First, the extracellular recordings reported here, and previously [17], demonstrate that the discharge profiles of multiple medullary and mesopontine nuclei are tightly coupled to behavioral state. Second, it appears that infant sleep is not mediated by distinct neurophysiological mechanisms, but rather by the very medullary and mesopontine nuclei that participate in sleep expression in adults. Such findings are not easily reconciled with numerous previous statements concerning the ontogeny of mammalian sleep and render moot attempts to explain sleep ontogeny as the gradual emergence of a coherent state from a set of undifferentiated, distinct, diffuse, or primitive sleep mechanisms [1,7,8,9]. In contrast, we have argued that the development of sleep entails the elaboration of an elementary sleep circuit that is largely in place soon after birth in rats [14]; accordingly, sleep components that emerge during ontogeny do so by integrating with those components that already exist. Of course, significant differences exist between infant and adult sleep and should not be discounted; for example, perhaps the most striking difference between infant and adult sleep is the rapidity with which infants cycle between sleep and wakefulness [19,64].

Establishing the developmental relations between sleep in infants and adults has important implications for theories concerning the functions of sleep. Many such theories can be applied to infants only with difficulty, because they address behaviors or contexts that are specific to adults. In addition, some theories place particular emphasis on the cerebral cortex [65,66], a part of the brain that, as mentioned above, does not exhibit state-dependent activity at those ages when infants are thought to sleep the most (e.g., before P12 in rats). If infant and adult sleep were as mechanistically distinct as some have argued, then one might imagine that sleep serves qualitatively different functions at different ages. But, as shown here, the basic mechanisms of infant and adult sleep are not distinct—even at ages before state-dependent neocortical activity is expressed—suggesting that there may be fundamental similarities between the functions of infant and adult sleep that have largely been overlooked. Thus, we believe that obtaining a clearer understanding of the mechanisms of sleep in infants is central to the goal of developing a theory of sleep that has applicability across the lifespan and can account for the inordinate amount of sleep during infancy [67].

## Materials and Methods

### 

All experiments were performed in accordance with National Institutes of Health guidelines for the care of animals in research and were approved by the Institutional Animal Care and Use Committee of the University of Iowa. All efforts were made to minimize the number of animals used and their suffering.

#### Subjects

A total of 64 P6–P10 Sprague-Dawley Norway rats *(Rattus norvegicus)* of both sexes from 46 litters were used. Litters were culled to 8 pups within 3 d of birth (day of birth = day 0). Mothers and their litters were housed in standard laboratory cages (48 × 20 × 26 cm) in the animal colony at the University of Iowa where food and water were available ad libitum. All animals were maintained on a 12:12 h light-dark schedule with lights on at 07:00 h. All experiments were conducted during the lights-on phase.

#### Retrograde tracing

Ten P8 rats from four litters were used (body weights, 16.1–22.9 g). While the pup was anesthetized with isoflurane, the head was secured in a stereotaxic instrument (David Kopf Instruments, Tujunga, California, United States) and leveled between bregma and lambda. A small burr hole was drilled over the target area and the retrograde tracer DiI (Molecular Probes, Eugene, Oregon, United States; 20 nl of 5% DiI in DMSO) was infused at the rate of 1nl/s using a microsyringe (model 7000.50C, Hamilton, Reno, Nevada, United States). Infusions were localized to the medial medulla (*n* = 5) and immediately lateral to it (*n* = 5), as described previously [17]. Coordinates relative to lambda for the medial site were AP, −2.5 mm; ML, 0.0 mm; DV, −12.3 mm; for the lateral site, the coordinates were AP, −2.5 mm; ML, 1.0 mm; DV, −12.3 mm. After the infusion was complete, the syringe remained in place for 5 min, was then retracted slowly, and the wound closed using cyanoacrylate adhesive.

After surgery, pups recovered in a warm, humidified incubator for 1–2 h before being returned to the home cage. After 26–28 h, the pups were overdosed with sodium pentobarbital (approximately 100 mg/kg intraperitoneally) and perfused transcardially with physiological saline followed by 3% formalin. Brains were postfixed in the skull for 1–2 d in a formalin-sucrose solution and then removed from the skull and fixed for at least two more days in a fresh solution. After fixation, the brains were sliced in 50-μm coronal sections with a sliding microtome (model SM 2000 R, Leica, Bensheim, Germany). The slices were mounted and coverslipped with Vectashield (Vector Laboratories, Burlingame, California, United States) and stored at 2–4 °C until inspected and photographed under fluorescent light using a digital microscope (model BX-51, Olympus, Japan).

Sections were visually inspected throughout the pontomedullary axis, and seven areas were selected for cell counting (Table 1). Next, digital photographs of one representative section of all seven structures were obtained from the same coronal level from each subject. Every effort was made to ensure that the cell counts were performed at the same level in each brain (roughly corresponding to plates 54, 55, 56, 58, and 66 in the adult rat brain atlas of Paxinos and Watson [68]). Finally, labeled cells were counted manually from each photo using ImageJ software (National Institutes of Health, Bethesda, MD).

#### Electrophysiology

Twenty-five P6–P10 rats from 20 litters were used (body weights, 13.4–25.7 g). Under isoflurane anesthesia, a precollicular decerebration was performed and bipolar nuchal EMG electrodes were implanted bilaterally. The infant's fragile skull was bleached, dried, and then coated with Vetbond (3M, St. Paul, Minnesota, United States) to add strength. Next, a custom-built, T-shaped, stainless steel head-plant, designed to attach to the earbar and nosebar holders of a stereotaxic instrument, was attached to the skull over the pretreated area using cyanoacrylate adhesive gel. After securing the head-plant and leveling the skull, a small hole was drilled over the recording site. Finally, to inhibit movement and calm the pup, it was wrapped in gauze [69] without obscuring movements of the tail.

Recordings were performed in a stereotaxic instrument with brain and body temperatures maintained at approximately 37 °C. Within 2–3 h of surgery, pups began exhibiting sleep-wake cycles, as judged by oscillations in muscle tone as well as myoclonic twitches against a background of nuchal atonia [19]. When sleep-wake cyclicity was observed, a stainless steel 8-trode (ALA Science, Westbury, New York United States), connected to a unity gain headstage and digital amplifier (Tucker-Davis Technologies, Alachua, Florida, United States), was lowered into the recording site while neurophysiological activity was monitored using an oscilloscope and audio analyzer (FHC, Bowdoinham, Maine, United States). The 8-trode consists of eight individual recording sites distributed about the tip (500–800 kΩ per electrode), with the entire electrode having a diameter of approximately 150 μm. The signals were amplified (10k) and filtered (500–5000 Hz band-pass) before being sampled at 12.5 kHz. When stable units were observed and at least one of these units appeared to exhibit state-dependent activity, the electrode was left in place for 5–10 min before data collection began. Neurophysiological and EMG data were recorded synchronously for 10 min (encompassing 4–12 sleep-wake cycles) to hard disk for off-line analysis. At the end of the experiment, the recording site was marked by passing a 50 μA anodal current through the eight closely spaced electrodes for 3 s (thereby producing a single small lesion).

In the current study, nuchal EMG was used as an indicator of behavioral state. As described previously [19], the EMG signal was dichotomized into bouts of high muscle tone and atonia (indicative of wakefulness and sleep, respectively). The sleep state was further categorized based on the presence or absence of phasic events (i.e., based on visual observation of myoclonic twitching of the tail, or based on twitching detected in the nuchal EMG). The reliable relationship between myoclonic twitching as assessed by behavioral observation or nuchal EMG has now been documented [62].

Four of the eight continuously recorded channels containing heterogeneous unit activity were selected for off-line analysis. Using Spike2 software (Cambridge Electronic Design, Cambridge, United Kingdom), a threshold was set for each of the four selected channels to extract spike data with a signal-to-noise ratio exceeding 2:1. Using the multichannel data, principal components analysis was then used for spike sorting [70,71]. The sorted units were assigned to groups using graphical cluster cutting [72,73]. To remove artificial clusters, spike waveforms were inspected and autocorrelations were constructed for each cluster. A cluster was deemed to contain a single unit only if the autocorrelation analysis indicated a refractory period of at least 2 ms.

Three types of analyses were performed. The first analysis was aimed at classifying neurons as either EMG-on or atonia-on. Periods of nuchal atonia and high muscle tone were identified from the EMG record, as described above. Next, the firing rates of individual sorted units were compared with the behavioral state of the animal across 4–12 pairs of high tone and atonia periods. State-related differences in mean discharge rates were tested using the Wilcoxon matched-pairs signed-ranks test [74]; alpha was set at 0.05. Neurons that significantly increased their firing rates during periods of high muscle tone were designated as EMG-on, and neurons that significantly increased their firing rates during atonia periods were designates as atonia-on.

The second analysis was aimed at determining whether discharge rates differed between sleep bouts that did or did not contain myoclonic twitching. Discharge rates during twelve 4-s segments of atonia with less than or equal to one nuchal EMG spike (indicative of sleep without phasic processes; i.e., QS), and discharge rates during twelve 4-s segments of atonia with more than four nuchal EMG spikes (indicative of sleep with phasic processes; i.e., active sleep, AS) were compared using the Wilcoxon matched-pairs signed-ranks test; alpha was set at 0.05.

The third analysis was aimed at revealing the relationship between the nuchal EMG twitches and neuronal burst activity. To this end, an event marker was placed on the center of all nuchal EMG twitches that occurred during a recording session. Next, a peristimulus histogram of unit activity triggered on the event marker was generated. Neurons were classified as twitch-on if the average discharge rate increased at least 4-fold before a twitch (measured 0–20 ms and 40–60 ms before a twitch).

For all experiments presented here, means are presented with their standard errors.

Brains were prepared for histological analysis as described above. After fixation, the brains were sliced in 25–50-μm sections with a sliding microtome and stained with cresyl violet. The locations of the marking lesions were determined by examining serial sections.

#### Lesions

Chemical lesions of the SubLC and PO were performed in 16 P8 nondecerebrated rats from ten litters (body weights, 17.7–23.9 g). A pup was anesthetized with isoflurane and its head was secured in a stereotaxic instrument, and the skull was leveled between bregma and lambda. A small burr hole was drilled over the target area and a microsyringe (model 7000.50C, Hamilton) was lowered stereotaxically either into the SubLC (*n* = 4; coordinates relative to lambda: AP, −1.8 mm; ML, ± 1.0 mm; DV, −11.8 mm) or into the PO (*n* = 5; coordinates relative to lambda: AP, −1.2 mm; ML, ± 1.0 mm; DV, −12.3 mm). Lesions were produced by infusing a high concentration of quisqualic acid (50 mM; 150 nl over 15 s) as described previously [17]. Control animals were treated identically but received infusions of Ringers solution only into the PO (*n* = 5) and SubLC (*n* = 2).

Lesions of the DLPT were performed in 13 P8 nondecerebrated rats from 12 litters (body weights, 17.4–21.8 g). Because previous attempts to use ibotenic acid in the brainstem of pups resulted in very high rates of mortality, and attempts to use quisqualic acid to lesion the DLPT were unsuccessful, electrolytic lesions were used here. After the pup was prepared for stereotaxic surgery, as described above, a concentric, bipolar, tungsten electrode (1 MΩ, 3–4 μm at the tip, model TM33CCINS, World Precision Instruments, Sarasota, Florida, United States) connected to a stimulus isolator (model A395, World Precision Instruments) and a stimulus generator (model 48, Grass, Quincy, Massachusetts, United States) was lowered into the PPT/LDT area (coordinates relative to lambda: AP, −0.8 mm; ML, ± 0.8 mm; DV, −4.4 mm). Lesions were produced by applying a 150-μA current for 15–30 s (*n* = 7). Control animals received identical treatment but without the current (*n* = 6).

After a chemical or electrolytic lesion was produced, bipolar nuchal EMG electrodes were implanted bilaterally, the pup was placed on a felt pad in a supine position (to enable observation of twitching in the individual limbs), and lightly restrained as described previously [4]. Next, the pups recovered in a humidified incubator (35 °C) for 3 h. After recovery, pups were intubated and infused with 2–4% of their body weight with warm cream and transferred to a humidified recording chamber maintained at 35 °C. A microcamera placed above the lid of the recording chamber allowed recording of sleep-wake behaviors, as described elsewhere [4]. Video data were recorded using a digital video system (model DV8, Vetron, Rebersburg, Pennsylvania, United States). After 1 h of acclimation to the chamber, synchronized behavioral and EMG data were collected for 1 h and recorded to digital tape for off-line scoring and analysis.

EMG signals were digitized at 5 kHz using a data acquisition system (BioPac Systems, Santa Barbara, California, United States). Next, the EMG signals were summed, integrated, and full-wave rectified, and mean bout durations of high-tone and atonia periods were measured over 60 min, as described previously [19]. Next, a trained observer viewed a 15-min taped segment of the pup's behavior and, using an event recorder, pressed a key when myoclonic twitches were detected [4]. Myoclonic twitches are phasic, rapid, and independent movements of the limbs and tail; high-amplitude movements comprise such wake-related behaviors as stretching, kicking, and yawning [75,76]. We and others have used similar scoring procedures in the past and have found them to be highly reliable, with inter- and intrarater reliability coefficients typically exceeding 0.85 [77,78]. The total number of twitches during the 15-min scoring session was determined, as well as the number of twitches that occurred against a background of high muscle tone.

For the DLPT lesion experiment, group differences in the expression of sleep components were assessed using an unpaired t-test. For the SubLC and PO lesion experiment, group differences were assessed using a single-factor analysis of variance (ANOVA); the post hoc test was Fisher's PLSD. For all tests, alpha was set at 0.05.

Histological methods were identical to those described above.

## Abbreviations

AS: active sleep
DLPT: dorsolateral pontine tegmentum
DR: dorsal raphé
EEG: electroencephalogram
EMG: electromyogram
Gi: nucleus gigantocellularis
LC: locus coeruleus
LDT: laterodorsal tegmental nucleus
LPB: lateral parabrachial nucleus
MIA: medullary inhibitory area
MPB: medial parabrachial nucleus
P[number]: postnatal day [number]
PC: nucleus pontis caudalis
PO: nucleus pontis oralis
PPT: pendunculopontine tegmental nucleus
QS: quiet sleep
REM: rapid eye movement
SLD: sublaterodorsal nucleus
SubLC: subcoeruleus
